# Establishing reference values for aortic dimensions in the general Indian population and implications for global standards

**DOI:** 10.1007/s12055-025-02173-6

**Published:** 2026-02-24

**Authors:** Mohammed Idhrees, Nora Bacour, Onur Dolmaci, Jedidiah Samraj, Bashi Velayudhan, Nimrat Grewal

**Affiliations:** 1 Institute of Cardiac and Aortic Disorders (ICAD), SRM Institutes for Medical Science (SIMS Hospital), Chennai, India; 2 Department of Cardiothoracic Surgery, Amsterdam University Medical Center, Meibergdreef 9, Amsterdam, 1105 AZ the Netherlands; 3 Department of Cardiology, OLVG, Amsterdam, the Netherlands; 4 Department of Cardiothoracic Surgery, Leiden University Medical Center, Leiden, the Netherlands; 5 Aortic Institute at Yale-New Haven Hospital, Yale University School of Medicine, New Haven, USA

**Keywords:** Aortic dimensions, Indian population, Ethnic differences, Cardiovascular health, Aortic diseases

## Abstract

**Background:**

The dimensions of the aorta play a crucial role in cardiovascular health, serving as a determinant of normalcy and a marker for pathological conditions such as aortic aneurysms and dissections. Extensive research has been conducted on the ascending aortic dimensions in the Western population, which forms the basis of the current aortic surgical guidelines. However, there remains a paucity of data specifically focused on the Indian population, which is essential for accurate diagnosis, risk stratification, and management of aortic diseases in the Indian context. This paper aims to describe the general aortic size distribution in the general Indian population.

**Methods:**

To study the aortic size in the Indian general population, we included all consecutive individuals who underwent a computed tomography (CT) scan between January 2022 and December 2022. Patients under the age of 18 years, those with cardiovascular comorbidities, and those with any aortic segment measuring > 45 mm were excluded from the analysis. The maximum aortic diameters were measured at predefined levels (aortic root, ascending aorta, aortic arch, proximal descending aorta, and the abdominal aorta).

**Results:**

Our study comprised 891 individuals with a mean age of 51.9 years, of which 41.5% were female. The mean ascending aortic diameter of all subjects was 29.3 ± 3.9 mm and was significantly smaller in women (28.7 ± 4.0 mm) as compared to men (29.8 ± 3.8 mm), even after correcting for age, body mass index (BMI), and body surface area (BSA). The proportion of subjects with an ascending aorta < 3.5 cm was 93.5%, that of subjects with 3.5–3.9 cm was 6.2%, and that of subjects with 4.0–4.4 cm was 0.3%.

**Conclusions:**

The general aortic dimension in the Indian population is deceptively small, most commonly < 3.5 cm. The ascending aorta is significantly smaller in the female population, also after correcting for age, BMI, and BSA. This study provides evidence to question current recommendations in the aortic guidelines for surgical intervention at 5–5.5 cm for the Indian population and whether a distinction should be made for the female patients.

**Graphical Abstract:**

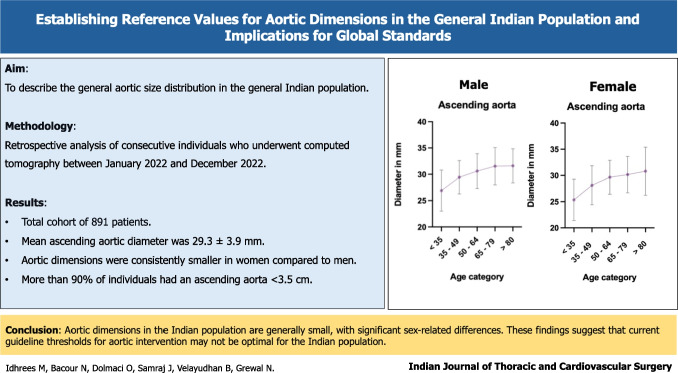

**Supplementary Information:**

The online version contains supplementary material available at 10.1007/s12055-025-02173-6.

## Introduction

Thoracic aortic aneurysms and dissections (TAADs) form a leading cause of death worldwide, with the majority of deaths being preventable if individuals at risk are identified and properly managed [1]. Despite advances in diagnostic methods, TAAD can be challenging to identify because its symptoms resemble those of other acute chest and abdominal conditions. Moreover, an aneurysmal disease, which forms a substantial risk factor for TAAD, often develops without noticeable symptoms, leaving many individuals unaware of their risk.

Currently, the primary method of prevention involves identifying individuals with aortic dilation at risk of dissection and considering surgical replacement as a preventative measure. However, accurately predicting who is at risk of aortic dissection is challenging. While conditions like hypertension and certain genetic syndromes can increase the likelihood of developing aortic aneurysms and dissections, pinpointing the occurrence of aortic dissection remains difficult [2–4]. Current aortic surgical guidelines provide comprehensive evidence-based recommendations for the diagnosis, management, and perioperative care of patients with aortic diseases, including aortic aneurysms, dissections, and other related conditions [5, 6]. By adhering to evidence-based practices outlined in these guidelines, healthcare providers can optimize patient outcomes, minimize complications, and improve the overall quality of care for individuals with aortic pathologies. Existing recommendations, which suggest replacing the ascending aorta when it reaches 5.5 cm in diameter for the general population, are largely based on expert opinions and retrospective data [5, 7, 8]. Furthermore, studies conducted in Western countries have established reference values for aortic dimensions through population-based cohorts [9, 10], which lay the foundation of the recommendations in the current aortic guidelines. However, the aorta exhibits considerable variability in size and morphology among different populations [11]. Therefore, there is growing recognition of the need to establish population-specific reference ranges to account for ethnic and geographical variations.

In India, where cardiovascular diseases are a significant health burden [12], understanding general aortic dimensions is essential for effective clinical practice. No research pertaining to the general aortic dimensions on cross-sectional imaging has been published so far in the adult Indian population. The objective of this study was therefore to perform an observational analysis of the thoracic aortic size distribution at multiple levels using computed tomographic data and plot reference curves.

## Methods

### Study population

This retrospective study was conducted at SRM Institutes for Medical Science (SIMS Hospital) in Chennai, India. Approval for this study was granted by the medical ethics committee of the SIMS Hospital, and patient consent was waived. Moreover, this study has been performed in accordance with the ethical standards as laid down in the Declaration of Helsinki. To generate an aortic size distribution of the general population using cross-sectional imaging, we chose to include all consecutive individuals—totaling 2000 patients—who underwent computed tomography (CT) between January 2022 and December 2022.

### Study parameters

The patients’ electronic health records were examined to obtain data regarding patient demographics and medical history (i.e., stroke, chronic kidney disease, peripheral arterial occlusive disease, chronic obstructive pulmonary disease). Cardiovascular risk factors, comorbidities, usage of tobacco, the body mass index (BMI), and the Du Bois body surface area (BSA) were scored for each patient [13]. Patients under the age of 18 years, those with cardiovascular comorbidities (hypertension, diabetes mellitus, chronic kidney disease, dyslipidemia, history of stroke, peripheral vascular disease, or known tobacco usage), and those with any aortic segment measuring > 45 mm were excluded from the study. Aortic diameters were obtained using CT imaging, as described in the following section.

### Computed tomography imaging

Non-contrast, non-electrocardiography (ECG)-gated CT scan images of each patient were analyzed by two independent researchers (MI and JS). No significant inter-observer variability was noted. Maximum aortic diameters were measured at predefined levels (aortic root, ascending aorta, aortic arch, proximal descending aorta, and the abdominal aorta). As described earlier by Elefteriades et al. [14], all diameter measurements were taken perpendicular to the long axis of the aorta at the point of maximal dilation. The aortic root was measured from sinus to sinus, the maximum ascending aortic diameter was measured manually from the largest portion of the ascending aorta up to the leading edge of the innominate artery, and the maximum arch diameter was measured between the innominate artery and the left subclavian artery. Maximal proximal aortic diameter was identified by measurements taken manually from the largest portion of the descending thoracic aorta beginning at the left subclavian artery and ending at the diaphragm. The abdominal artery was measured at the level of the renal artery.

### Statistical analysis

In this study, skewness, kurtosis, and visual-normality tests (histograms and *Q*–*Q* plots) were performed for all variables. Normally distributed continuous variables are presented as mean ± standard deviation (SD), while continuous variables with a non-normal distribution are presented as median and interquartile range (IQR). Categorical data are presented as frequencies and percentages.

To analyze the baseline characteristics and aortic diameters between males and females, an unpaired *T*-test was used in case of normally distributed data, while a Mann-Whitney *U* test was used for non-normally distributed data. Categorical data was analyzed using Fisher’s exact test. To further analyze the aortic diameters, a multivariable linear regression analysis was performed, correcting for clinically relevant variables: sex, age, BMI, and BSA. A *p*-value of < 0.05 was considered to be significant. All statistical analyses were conducted using IBM SPSS version 30.0. GraphPad software was used to create Figs. 2 and 3.

## Results

### Baseline characteristics

A total of 2000 individuals who underwent CT scanning between January 2022 and December 2022 were included in the study. After excluding patients with cardiovascular comorbidities and those with any aortic segment measuring > 45 mm, a total of 891 patients were included in the study, comprising 370 female (41.5%, mean age 52.9 years) and 521 male (58.5%, mean age 51.2 years). In the cohort, female subjects were significantly shorter and had a smaller BSA compared to male subjects (*p* < 0.001 and *p* = 0.007, respectively). The median blood pressure did not differ significantly between sexes (both *p* > 0.05). Baseline characteristics are summarized in Table 1. Characteristics of the total cohort, including those with comorbidities, are presented in supplementary Table 1.

**Table 1:** Baseline characteristics

Characteristic	Total *N* = 891	Male *N* = 521	Female *N* = 370	*p*-value
Age (in years)	51.9 ± 17.7	51.2 ± 17.6	52.9 ± 17.7	0.147
Height (in cm)	160.1 ± 6.3	161.1 ± 6.5	158.8 ± 5.7	< 0.001
Weight (in kg)	61.6 ± 8.8	62.6 ± 8.8	60.3 ± 8.6	< 0.001
BMI	23.9 ± 2.8	24.0 ± 2.6	23.9 ± 2.9	0.630
BSA	1.65 ± 0.2	1.67 ± 0.2	1.64 ± 0.2	0.007
Arterial blood pressure in mmHg Systolic Diastolic	130 (120–130) 90 (80–90)	130 (120–130) 90 (80–90)	130 (120–130) 90 (80–90)	0.718 0.704

BMI= body mass index (in kg/m^2^); BSA= body surface area (in m^2^)

Data are presented as mean ±  standard deviation (SD) or median (interquartile range)

### Aortic dimensions

**Fig. 1:**
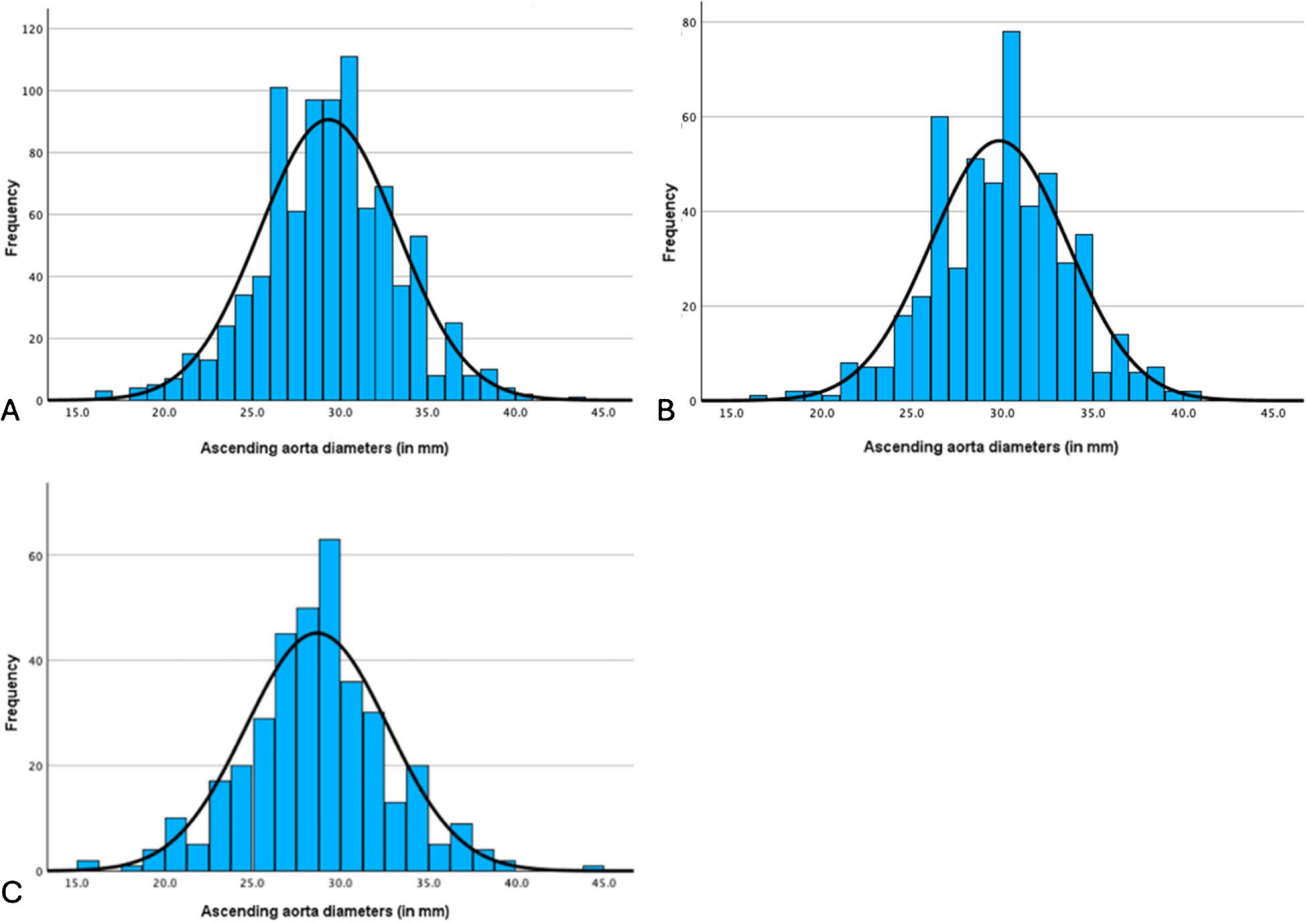
Ascending aortic diameter distribution. The ascending aortic dimension distribution is shown in the total population (**A**), in the male individuals (**B**), and female individuals (**C**).

The aortic dimensions are displayed in Table 2. The mean aortic root diameter was 31.5 ± 3.8 mm in female and 33.9 ± 3.9 mm in male subjects (*p* < 0.001). The mean ascending aortic diameter was also significantly smaller in female compared to male individuals, 28.7 ± 4.0 mm vs 29.8 ± 3.8 mm (*p* < 0.001) (Fig. 1). The mean aortic arch diameter was 25.0 ± 3.4 mm in female and 25.7 ± 3.2 mm in male (*p* < 0.001). Mean proximal descending aortic dimensions were 21.1 ± 3.3 mm in female and 22.6 ± 3.5 mm in male (*p* < 0.001), and the mean abdominal aortic diameter was 19.7 ± 3.1 mm in female and 21.1 ± 3.1 mm in male (*p* < 0.001). The aortic dimensions of the total cohort, including those with comorbidities, are presented in supplementary Table 2.

**Table 2:** Aortic diameters

Characteristic	Total *N* = 891	Male *N* = 521	Female *N* = 370	*p*-value
Sinus of Valsalva (mm) Minimum Maximum Mean (+ SD) Median (IQR)	18.0 44.5 32.9 ± 4.1 32.7 (30.2–35.9)	18.0 44.5 33.9 ± 3.9 34.1 (31.3–36.5)	18.0 41.8 31.5 ± 3.8 31.3 (29.1–34.2)	< 0.001
Sinotubular junction (mm) Minimum Maximum Mean (+ SD) Median (IQR)	14.5 40.1 25.8 ± 3.4 25.9 (23.4–28.1)	14.5 38.9 26.3 ± 3.2 26.4 (24.2–28.5)	15.1 40.1 25.0 ± 3.7 24.8 (22.5–27.3)	< 0.001
Ascending aorta (mm) Minimum Maximum Mean (+ SD) Median (IQR)	16.0 43.8 29.3 ± 3.9 29.3 (26.7–31.9)	16.0 40.2 29.8 ± 3.8 30.0 (27.0–32.4)	16.0 43.8 28.7 ± 4.0 28.8 (26.4–30.8)	< 0.001
Aortic arch (mm) Minimum Maximum Mean (+ SD) Median (IQR)	12.7 43.1 25.4 ± 3.3 25.6 (23.4–27.6)	16.2 43.10 25.7 ± 3.2 26.1 (24.0–28.0)	12.7 37.7 25.0 ± 3.4 25.0 (22.8–27.1)	< 0.001
Descending thoracic aorta (mm) Minimum Maximum Mean (+ SD) Median (IQR)	10.9 39.5 22.0 ± 3.5 21.8 (19.7–24.1)	10.9 36.8 22.6 ± 3.5 22.2 (20.3–24.6)	13.6 39.5 21.1 ± 3.3 20.9 (18.9–23.2)	< 0.001
Abdominal aorta (mm) Minimum Maximum Mean (+ SD) Median (IQR)	12.1 36.6 20.5 ± 3.2 20.2 (18.7–22.3)	12.6 36.6 21.1 ± 3.1 20.7 (19.1–22.8)	12.1 31.3 19.7 ± 3.1 19.7 (18.0–21.3)	< 0.001

SD= standard deviation; IQR= interquartile range

**Fig. 2:**
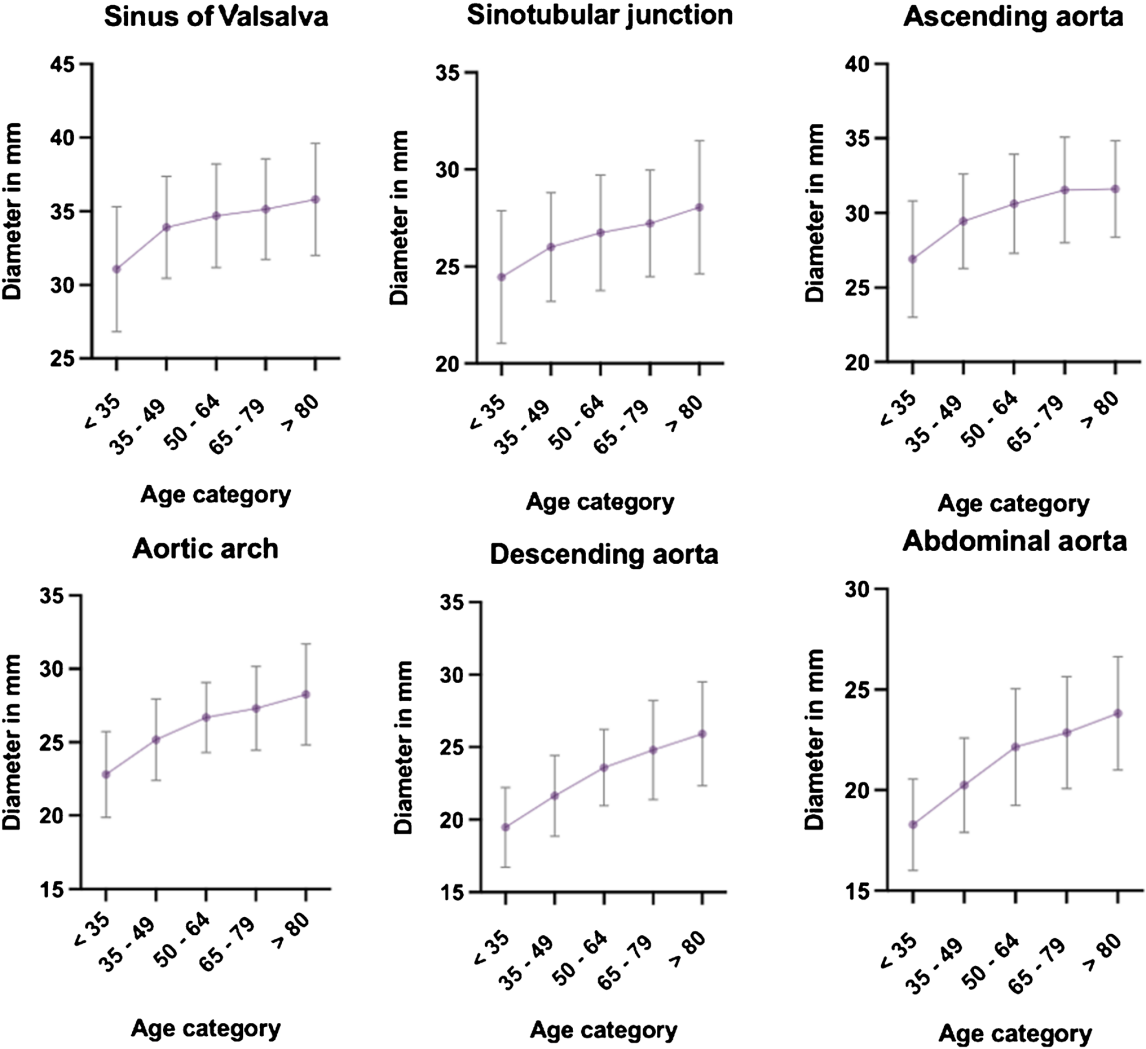
Aortic dimensions in male individuals. Distribution (mean ± standard deviation) of the aortic dimensions at the level of the sinus of Valsalva, the sinotubular junction, ascending aorta, aortic arch, descending aorta, and abdominal aorta plotted against age in male individuals.

**Fig. 3:**
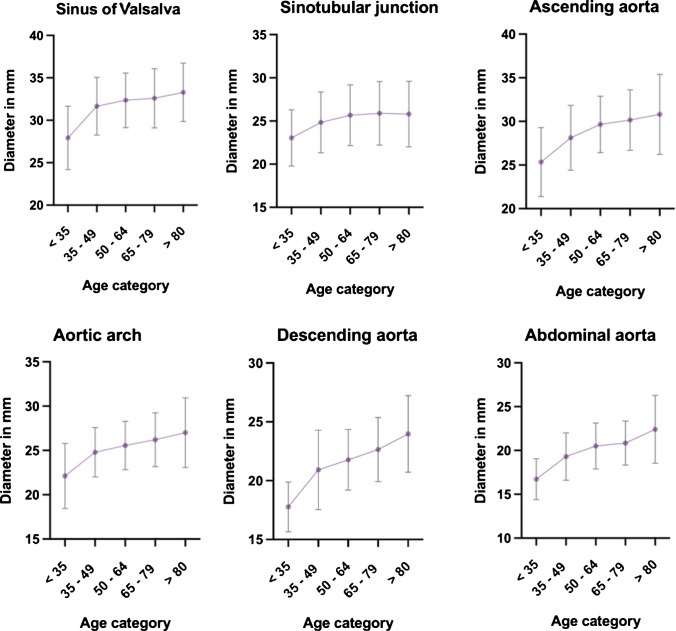
Aortic dimensions in female individuals. Distribution (mean ± standard deviation) of the aortic dimensions at the level of the sinus of Valsalva, the sinotubular junction, ascending aorta, aortic arch, descending aorta, and abdominal aorta plotted against age in female individuals.

After adjusting for age, BMI, and BSA, sex differences in aortic diameter remained statistically significant across all measured segments, with lower diameters in females: sinus of Valsalva, unstandardized beta (*B*) = −2.555 mm, 95% confidence interval (CI) –3.035 to −2.074; *p* < 0.001; sinotubular junction, *B* = −1.285 mm, 95% CI −1.716 to −0.855; *p* < 0.001; ascending aorta, *B* = −1.274 mm, 95% CI −1.743 to −0.804; *p* < 0.001; aortic arch, *B* = −0.898 mm, 95% CI −1.279 to −0.517; *p* < 0.001; descending thoracic aorta, *B* = −1.666 mm, 95% CI −2.043 to −1.290; *p* < 0.001; and abdominal aorta, *B* = −1.557 mm, 95% CI −1.909 to −1.205; *p* < 0.001 (Table 3). Figures 2 and 3 give an overview of the aortic diameters across the aortic sinus, sinotubular junction, ascending aorta, aortic arch, and descending aorta plotted against age in male and female individuals respectively. The adjusted aortic dimensions of the total cohort, including those with comorbidities, are presented in supplementary Table 3.

**Table 3:** Multivariate linear regression model

Predictor	B (95% CI)	SE	T	*P*-value
Sinus of Valsalva				
Constant	26.863 (24.349— 30.202)	1.185	20.972	<0.001
Age	0.086 (0.072–0.107)	0.007	12.605	<0.001
Female	−2.555 (−3.035—−2.074)	0.245	−10.429	<0.001
BMI	−0.027 (−0.132—0.078)	0.054	−0.502	0.616
BSA	1.990 (0.345–3.635)	0.838	2.374	0.018
Sinotubular junction				
Constant	20.786 (18.536—23.036)	1.146	18.131	<0.001
Age	0.059 (0.047—0.071)	0.006	9.732	<0.001
Female	−1.285 (−1.716—−0.855)	0.219	−5.864	<0.001
BMI	0.038 (−0.056—0.132)	0.048	0.792	0.429
BSA	0.920 (−0.553—2.392)	0.750	1.226	0.221
Ascending aorta				
Constant	22.830 (20.374—25.287)	1.252	18.241	<0.001
Age	0.096 (0.083—0.109)	0.007	14.422	<0.001
Female	−1.274 (−1.743—−0.804)	0.239	−5.321	<0.001
BMI	0.016 (−0.087—0.119)	0.052	0.309	0.758
BSA	1.012 (−0.595—2.620)	0.819	1.236	0.217
Aortic arch				
Constant	17.179 (15.187—19.172)	1.015	16.925	<0.001
Age	0.092 (0.082—0.103)	0.005	17.084	<0.001
Female	−0.898 (−1.279—−0.517)	0.194	−4.624	<0.001
BMI	0.079 (−0.004—0.163)	0.042	1.869	0.062
BSA	1.164 (−0.139—2.468)	0.664	1.753	0.080
Descending aorta				
Constant	14.133 (12.166–16.101)	1.002	20.507	<0.001
Age	0.109 (0.099—0.120)	0.005	17.195	<0.001
Female	−1.666 (−2.043—−1.290)	0.192	−8.692	<0.001
BMI	0.074 (−0.008—0.156)	0.042	1.764	1.039
BSA	0.682 (−0.606 – 1.969)	0.656	1.039	0.299
Abdominal aorta				
Constant	14.152 (12.312—15.991)	0.937	15.100	<0.001
Age	0.096 (0.086—0.105)	0.005	19.210	<0.001
Female	−1.557 (−1.909—−1.205)	0.179	−8.688	<0.001
BMI	0.053 (−0.024—0.130)	0.039	1.354	0.886
BSA	0.477 (−0.726—1.681)	0.613	0.778	0.176

BMI = Body Mass Index (in kg/m^2^), BSA = Body Surface Area (in m^2^), B = unstandardized beta, CI =confidence interval

### Ascending aortic dimension distribution

As the surgical guidelines have described aortic management according to the ascending aortic diameter, we sought to analyze the percentage of individuals with an ascending aortic diameter below the described threshold. The distribution of the ascending aortic diameter across various size categories has been described in Table 4. The proportion of subjects with an ascending aorta ≤ 3.4 cm was 93.5%, that of subjects with 3.5–3.9 cm was 6.2%, and that of subjects with ≤ 4.4 cm was 0.3%.

**Table 4:** Ascending aortic diameter distribution

Aortic diameter in mm	Total *N* = 891	Male *N* = 521	Female *N* = 370	*p*-value
≤ 24	105 (11.8)	46 (8.8)	59 (15.9)	0.008
25–34	728 (81.7)	438 (84.1)	290 (78.4)	
35–39	55 (6.2)	35 (6.7)	20 (5.4)	
≤ 44	3 (0.3)	2 (0.4)	1 (0.3)	

Data are presented as *n* (%)

## Discussion

The findings of this study shed light on the range of aortic dimensions in the general Indian population, filling a significant gap in the existing literature. The size of the aorta is a crucial determinant of cardiovascular health, serving as a marker for pathological conditions such as aortic aneurysms and dissections. While extensive research has been conducted on aortic dimensions in the Western populations, there has been a lack of data specifically focused on the Indian population (being the most populous country, contributing over 17% of the world’s population).

A notable finding from our study is the smaller size of the ascending aorta in the Indian population as compared to earlier published data of mostly Caucasian individuals. Wolak et al. studied a United States (US) population who underwent coronary artery calcium scanning and described a mean ascending aortic size of 31.8 ± 3.7 in females and 34.0 ± 4.1 in males [15].

Another study conducted in the general population of the US by Paruchuri et al. describes the mean diameter of the ascending aorta of 3.2 cm [9]; however, differences between sexes were not investigated [10, 16]. The smaller average ascending aortic diameter of 29.3 mm observed in our study population thus underscores the importance of considering population-specific factors when interpreting aortic dimensions. Furthermore, in our study, significant differences were observed between sexes, with women consistently exhibiting smaller aortic dimensions compared to men, even after adjusting for age, BMI, and body size.

Based on the Wolak et al. study, the average ascending aorta size for males is 34.0 mm and for females is 31.8 mm. When extrapolated to the average BSA of an American, the aortic size index is as follows: 17.89 mm/m^2^ for males (BSA 1.9 m^2^) and 17.67 mm/m^2^ for females (BSA 1.8 m^2^) [15].

Our study reveals that, using the mean BSA of 1.67 m^2^ in men and 1.64 m^2^ in women, the aortic size indices are 17.80 mm/m^2^ for males and 17.50 mm/m^2^ for females. These values highlight that while the absolute aortic sizes are smaller compared to the Western population, the indices are similar. This observation reinforces the importance of considering BSA when assessing aortic dimensions, as population-specific differences play a critical role in clinical interpretation.

Therefore, we believe that the aortic size index is a more appropriate measure than absolute size. A study by Davies et al. [17] adds to this statement by suggesting that relative aortic size is more important than absolute aortic size in assessing the risk of complications. Previous research also indicates that a simpler height-based ratio, without the need for weight and BSA calculations, provides sufficient predictive value for adverse aortic events [18]. However, further research is needed to validate these conclusions.

These findings have several implications for clinical practice and research. Firstly, they highlight the need for tailored approaches to the diagnosis and management of aortic diseases in the Indian population. Current surgical guidelines, which are primarily based on data from Western populations, may need to be reevaluated to account for the smaller aortic dimensions observed in Indian individuals [5, 6]. These guidelines should also examine whether smaller aortic sizes, as found within the Indian population in our cohort, are associated with the risk of complications, such as dissection or rupture. Lastly, the observed gender differences in aortic dimensions underscore the importance of considering gender-specific factors in risk assessment and treatment planning for aortic diseases.

## Limitations

While our study provides valuable insights into aortic dimensions in the Indian population, it is not without limitations. Firstly, data analyzed in our study was uni-centric and retrospectively obtained, which could influence our findings. Furthermore, non-contrast, non-ECG-gated CT scans were utilized in our study, limiting the accuracy of the measurements. Future research endeavors should aim to include larger and more diverse study populations to validate our findings while exploring additional factors influencing aortic dimensions in the Indian context.

## Conclusion

In conclusion, our study contributes to a better understanding of aortic dimensions in the Indian population and emphasizes the importance of population-specific data in guiding clinical practice and research efforts aimed at improving cardiovascular health outcomes.

## Supplementary Information

Below is the link to the electronic supplementary material.

ESM 1 (DOCX 26.7 KB)

ESM 2 (MP4 18.0 MB)

## Data Availability

The datasets generated for this study can be made available upon reasonable request.
