# Supplementary material for: Establishing reference values for aortic dimensions in the general Indian population and implications for global standards
Source: Indian J Thorac Cardiovasc Surg. 2026 Feb 24;42(5):586–94. doi: 10.1007/s12055-025-02173-6 (PMC13065947; doi:10.1007/s12055-025-02173-6)
Supplement: Supplementary file 1 — (DOCX 26.7 KB) [file 12055_2025_2173_MOESM1_ESM.docx]

**Supplementary information**

Supplementary table 1: Baseline characteristics of all-comers

| Characteristic | Total N==2000 | Male  n=1224 | Female  n=776 | p-value |
| --- | --- | --- | --- | --- |
| Age (in years) | 61 (49-72) | 62 (49-72) | 61 (59-71) | 0.814 |
| Height (in cm) | 160 ± 6.0 | 161 ± 5.8 | 158 ± 6.0 | <0.001 |
| Weight (in kg) | 62.3 ± 9.5 | 63.1 ± 9.4 | 61.1 ± 9.6 | <0.001 |
| BMI | 24.2 ± 3.2 | 24.2 ± 3.0 | 24.2 ± 3.6 | 0.967 |
| BSA | 1.63 (1.63-1.73) | 1.63 (1.63-1.74) | 1.63 (1.59-1.69) | <0.001 |
| Arterial blood pressure in mmHg  Systolic  Diastolic | 130 (120-130)  90 (80-90) | 130 (120-130)  90 (80-90) | 130 (120-130)  90 (80-90) | 0.793  0.709 |
| Hypertension | 873 (43.65) | 535 (43.7) | 338 (43.6) | 0.963 |
| Diabetes Mellitus | 760 (38) | 489 (40) | 271 (34.9) | 0.026 |
| Dyslipidemia | 21 (1.05) | 13 (1.1) | 8 (1.0) | 1.000 |
| Chronic Kidney Disease | 79 (3.95) | 56 (4.6) | 23 (3) | 0.078 |
| Stroke | 20 (1) | 13 (1.1) | 7 (0.9) | 0.820 |
| PAOD | 3 (0.15) | 2 (0.2) | 1 (0.1) | 1.000 |
| Tobacco usage | 9 (0.45) | 7 (0.6) | 2 (0.3) | 0.496 |
| BMI = Body Mass Index (in kg/m^2^), BSA = Body Surface Area (in m^2^), COPD = Chronic Obstructive Pulmonary Disease, PAOD = Peripheral Arterial Occlusive Disease | | | | |

*Data are presented as mean ± SD (standard deviation) or median (interquartile range) or n (%)*

Supplementary table 2: Aortic diameters of all-comers

| Characteristic | Total  n=2000 | Male  n=1224 | Female  n=776 | p-value |
| --- | --- | --- | --- | --- |
| Sinus of Valsalva (mm)  Minimum  Maximum  Mean (+SD)  Median (IQR) | 18.0  85.7  33.9 ± 4.6  33.8 (30.8-36.6) | 18.0  85.7  35.1 ± 4.5  35 (32.4-37.5) | 18.0  82.4  32.1 ± 4.3  31.8 (30.0-34.6) | <0.001 |
| Sinotubular junction (mm)  Minimum  Maximum  Mean (+SD)  Median (IQR) | 14.5  69.7  26.6 ± 3.9  26.4 (24.2-28.7) | 14.5  69.7  27.2 ± 3.8  26.9 (24.8-28.9) | 15.1  40.1  25.6 ± 3.7  25.4 (22.9-28) | <0.001 |
| Ascending aorta (mm)  Minimum  Maximum  Mean (+SD)  Median (IQR) | 16.0  64.3  30.5 ± 4.3  30.2 (27.9-32.8) | 16.0  64.3  31.2 ± 4.3  30.9 (28.7-33.6) | 16.0  46.7  29.4 ± 4.1  29.2 (27-31.6) | <0.001 |
| Aortic arch (mm)  Minimum  Maximum  Mean (+SD)  Median (IQR) | 12.7  74.8  26.4 ± 3.7  26.4 (24.2-28.4) | 16.2  74.8  26.8 ± 3.8  26.7 (24.6-28.8) | 12.7  54.5  25.6 ± 3.5  25.8 (23.7-27.6) | <0.001 |
| Descending thoracic aorta (mm)  Minimum  Maximum  Mean (+SD)  Median (IQR) | 10.9  77.3  23.7 ± 4.1  23.6 (20.5-24.9) | 10.9  77.3  26.4 ± 3.7  26.4 (21.3-25.8) | 13.6  52.4  21.7 ± 3.2  21.7 (19.7-23.5) | <0.001 |
| Abdominal aorta (mm)  Minimum  Maximum  Mean (+SD)  Median (IQR) | 12.1  50.5  21.3 ± 3.4  20.8 (19.3-23.0) | 12.6  50.5  22.0 ± 3.4  21.9 (19.9-24.0) | 12.1  31.3  20.1 ± 2.8  20.1 (18.5-21.7) | <0.001 |
| SD = Standard Deviation, IQR = interquartile range | | | | |

Supplementary table 3: Multivariate linear regression model of all-comers

| Predictor | B (95% CI) | SE | T | P-value |
| --- | --- | --- | --- | --- |
| Sinus of Valsalva | | | | |
| Constant | 28.482 (26.158 - 30.807) | 1.185 | 24.028 | <0.001 |
| Age | 0.072 (0.061 - 0.086) | 0.006 | 11.777 | <0.001 |
| Female | -2.818 (-3.205 - -2.432) | 0.197 | -14.316 | <0.001 |
| BMI | -0.103 (-0.189 - 0.018) | 0.044 | -2.370 | 0.018 |
| BSA | 2.698 (0.778- 4.617) | 0.979 | 2.756 | 0.006 |
| Hypertension | 0.575 (0.139 -1.012) | 0.222 | 2.586 | 0.010 |
| Diabetes Mellitus | -0.112 (-0.555 – 0.332) | 0.226 | -0.493 | 0.622 |
| Sinotubular junction | | | | |
| Constant | 22.579 (20.767- 24.390) | 0.924 | 24.439 | <0.001 |
| Age | 0.049 (0.039 - 0.060) | 0.005 | 9.075 | <0.001 |
| Female | -1.580 (-1.913- -1.247) | 0.170 | -9.293 | <0.001 |
| BMI | 0.033 (-0.035 - 0.101) | 0.035 | 0.948 | 0.343 |
| BSA | 0.371 (-0.984 - 1.725) | 0.691 | 0.537 | 0.591 |
| Hypertension | 0.724 (0.343 – 1.105) | 0.194 | 3.725 | <0.001 |
| Diabetes Mellitus | -0.179 (-0.567 - 0.208) | 0.197 | -0.909 | 0.364 |
| Ascending aorta | | | | |
| Constant | 20.063 (19-891- 24.236) | 1.108 | 19.917 | <0.001 |
| Age | 0.080 (0.069 - 0.092) | 0.006 | 13.787 | <0.001 |
| Female | -1.666 (-2.027 - -1.305) | 0.184 | -9.054 | <0.001 |
| BMI | -0.037 (-0.117 - -0.043) | 0.041 | -0.906 | 0.365 |
| BSA | 2.918 (-1.124 – 4.712) | 0.915 | 3.190 | 0.001 |
| Hypertension | 0.944 (0.537 – 1.352) | 0.208 | 4.542 | <0.001 |
| Diabetes Mellitus | -0.116 (-0.530 – 0.299) | 0.211 | -0.548 | 0.584 |
| Aortic arch | | | | |
| Constant | 16.675 (14.807-18.544) | 0.953 | 17.501 | <0.001 |
| Age | 0.079 (0.069 - 0.088) | 0.005 | 15.692 | <0.001 |
| Female | -1.080 (-1.390 - - 0.770) | 0.158 | -6.824 | <0.001 |
| BMI | -0.003 (-0.072 - 0.066) | 0.035 | -0.078 | 0.938 |
| BSA | 3.198 (1.655 - 4.741) | 0.787 | 4.064 | <0.001 |
| Hypertension | 0.550 (0.199 - 0.901) | 0.179 | 3.074 | 0.002 |
| Diabetes Mellitus | -0.141(-0.498 - 0.215) | 0.182 | -0.777 | 0.437 |
| Descending aorta | | | | |
| Constant | 15.179 (13.270 - 17.088) | 0.973 | 15.593 | <0.001 |
| Age | 0.088 (0.078 - 0.098) | 0.005 | 17.195 | <0.001 |
| Female | -1.956 (-2.273 - -1.639) | 0.162 | -12.096 | <0.001 |
| BMI | -0.040 (-0.110 - 0.030) | 0.036 | -1.120 | 0.263 |
| BSA | 2.502 (0.925 - 4.078) | 0.804 | 3.112 | 0.002 |
| Hypertension | 0.898 (0.540 - 1.257) | 0.183 | 4.917 | <0.001 |
| Diabetes Mellitus | -0.655 (-1.019 - -0.291) | 0.186 | -3.527 | <0.001 |
| Abdominal aorta | | | | |
| Constant | 15.207 (13.585 - 16.828) | 0.827 | 18.396 | <0.001 |
| Age | 0.080 (0.071 - 0.088) | 0.004 | 18.330 | <0.001 |
| Female | -1.867 (-2.136 - -1.598) | 0.137 | -13.597 | <0.001 |
| BMI | -0.004 (-0.064 - 0.055) | 0.030 | -0.143 | 0.886 |
| BSA | 1.284 (-0.055 - 2.622) | 0.683 | 1.880 | 0.060 |
| Hypertension | 0.451 0.146 - 0.755) | 0.155 | 2.904 | 0.004 |
| Diabetes Mellitus | -0.388 (-0.697 - -0.078) | 0.158 | -2.459 | 0.014 |
| BMI = Body Mass Index (in kg/m^2^), BSA = Body Surface Area (in m^2^), B = unstandardized beta, CI =confidence interval | | | | |
